# Multilayer acoustic invisibility cloak based on composite lattice

**DOI:** 10.1038/s41598-022-20052-7

**Published:** 2022-09-27

**Authors:** Mansour Zaremanesh, Ali Bahrami

**Affiliations:** Optoelectronics and Nanophotonics Research Laboratory (ONRL), Faculty of Electrical Engineering, Sahand University of Technology, Tabriz, Iran

**Keywords:** Condensed-matter physics, Structural materials

## Abstract

A concentric cylindrical cloak is showed here to achieve the acoustic cloaking phenomenon. The introduced structure consists of MNE layers and water in MNE substrate in the MHz frequency range. Due to avoiding the incoming acoustic waves by the shell, the object can be hidden inside the cylindrical area of any shape. In order to improve the quality of cloaking, we have optimized the desired shell by considering the manufacturing technology. We show that an optimized, acoustic cloak based on composite lattice structure can reduce the scattering of an object more than a 20-layer realization of acoustic cloak based on multilayer cylindrical structure. This design approach can substantially simplify the fabrication of cloaking shells. In this research, to study the acoustic distribution of the desired structure, finite element method (FEM) has been used to analyze the structure in two dimensions and a cloak of natural materials with isotropic properties has been designed using effective medium theory**.**

## Introduction

Recently, invisibility and cloaking have been recognized as one of the most attractive fields of study and research. So far, various methods have been proposed to achieve invisibility and cloaking. Manipulation of synthetic materials such as metamaterials, phononic crystals, transformation optics, integration of surfaces and materials with a refractive index close to zero, carpet cloakings, etc., are examples of methods to achieve the cloaking^1–12^. The cloak can be achieved by controlling the sound (or light) flow and the phase front from microwave region to optical region using various methods such as phase control by metasurfaces, photonics and phononic crystals, unusual phenomena such as negative refraction, etc^13–15^. If the incident sound wave can bypass the object and is not penetrable and be able to remove the scattered sound, we will have the cloaking phenomenon. Controlling acoustic waves using synthetic materials is a significant issue in material physics. Pendry et al. proposed an invisible cloaking structure that could hide objects from electromagnetic radiation while cancelling external scattering based on the coordinate transformation theory^16^. Prototypes of many of the proposed electromagnetic shells were built based on metamaterial technology^17,18^. The cloaking concept then developed to the acoustic field and opened a new application for acoustic cloaking technology, and a similar method has been developed for designing acoustic shells. Cummer et al. showed a two-dimensional acoustic cloak from an acoustic material with mass anisotropy^19^. In this regard, Milton et al. Conceptually explain how spring masses can make mass anisotropy possible^20^. In another work on the physical realization of metamaterial with mass density anisotropy, the authors showed that such an unusual property could be made possible by using solid cylindrical asymmetric lattice^21^. Chen et al. approved the full 3D acoustic cloak^22^. The realization of the acoustic cloak depends on the elastic transformation medium, which must show radius-dependent distributions for anisotropic density and bulk modulus. One possible solution to this problem is to use acoustic crystals or acoustic metamaterials consisting of resonant elements below the wavelength. However, the acoustic metamaterial operates in a limited frequency range around its resonant frequency. The local resonance of its components may cause a significant absorption of the acoustic wave, which contradicts the concept of cloaking. The solution reported by Cummer for acoustic cloak requires a fluid material with anisotropic density and a scalar bulk modulus, and these parameters must depend on the radial distance to the object.

Inertial cloaking is made with anisotropic inertial properties. Pure pentamode cloaking is made of solids with anisotropic single elastic tensors^23^. However, the acoustic cloaking associated with inertial and pentamode cloaks includes mass and elastic anisotropy. Hasanpour et al. considered the interaction of an electromagnetic wave with a non-inertial cloak and, using a gyrating cloak, checked the effect of rotational speed on the scattering pattern and determined the direction of rotation and the magnitude of the rotational speed^24^, but they are associated with manufacturing restrictions. Also, Song et al. using acoustic metasurface, addressed the problems of band narrowness and specific incident angle for carpet cloaking^25^. Kim et al. investigated the use of an optimized structure as a double split hollow sphere acoustic cloaking structure for irregular surfaces^26^. Using acoustic illusions, Lin et al. provide the ability to misrepresent the sound field of an object^27^. Ahmed et al. present the notion of a machine learning driven acoustic cloak. Using neural networks, they restore the structural and material properties of the cloaking shell around the object, which suppresses the scattering of sound over a wide spectral range^28^. Fuji et al. used acrylonitrile butadiene styrene copolymers to minimize the scattering of airborne and waterborne sounds around acoustic cloaks. They optimized the designed acoustic cloakings using topology optimization based on the covariance matrix adaptation evolution strategy^29^. Using effective environment theory, Zhu et al. designed the cloak for underwater operation by using the multi-zone coordinate transformation method in order to overcome the difficulty of achieving ideal parameters for the construction of the cloak^30^. One of the most important limitations of the mentioned methods is that they work best for a single frequency or in several separate frequencies. The mentioned limitations restrict the possibility of realizing and making acoustic cloaking. Also, there are no materials with such unusual properties in nature. In order to obtain a material with anisotropic behavior, complex microstructures designed with homogenization-based optimization techniques have been used^2^. Numerous attempts have been made to overcome such limitations and facilitate a more straightforward design, for example, by using quasi-coherent cloaks in which the conversion is made explicitly in such a way as to ignore anisotropy in the distribution of the obtained materials^31^. However, the geometries that allow this technique is limited, and the shell must, in principle, include the available space. Therefore, they should introduce engineered materials that minimize the mentioned limitations. The design and arrangement of materials has an important role in determining the properties of internal and external waves of those materials.

Multilayer cylindrical cloaks have shown good potential as candidates for cloaking devices^32–34^. The structure presented in this paper goes beyond metamaterials in some respects. Despite metamaterial-based cloakings, which are only possible within a specific frequency range and have losses, multilayer cloakings made of isotropic materials are more appropriate to construct than the other methods. Due to the scalability of multilayer cloakings, these structures can be designed to operate in any desired wavelength range. In a multilayer cloaking structure, we can freely adjust how the wave propagates from the source and the distance of the structure without affecting the cloaking result.

In general, to improve the hiding performance, some key requirements must be met. For example, the feasibility and simplicity of fabricating structure and using the available materials are important. Also, the consistent performance of the system is essential for transverse and longitudinal shear waves, as well as no incident angle limitation in the cloaking phenomenon for the designed structure. The proposed scheme should be applicable to hide the object of any shape in the cloak area. Here, a multilayer cloaking is proposed to achieve a cloak region that meets the requirements listed above. This article is organized so that the next section, we examine the ideal structure to achieve the phenomenon of acoustic cloak and ahead, we introduce the structures, materials, and results of this research.

## The ideal structure of a cylindrical cloak

Recent studies have shown the possibility of constructing an ideal cloaking using materials with unusual properties. In these studies, a cylindrical cloak is designed to make the object vanish from acoustic waves. Using the same principle, the researchers designed a cloak to concealment an object from sound radiation using metamaterials^22,35,36^. This work investigates sound scattering from plane waves on the cylindrical cloaking structure. The material parameters for an ideal acoustic cylindrical cloaking are given below^19^:

1 $$ \begin{gathered} \rho_{1} = \frac{{r + \sqrt {2rR_{1} - R_{1}^{2} } }}{{r - R_{1} }}\rho_{b} \hfill \\ c_{1} = \frac{{R_{2} - R_{1} }}{{R_{2} }}\frac{r}{{r - R_{1} }}c_{b} \hfill \\ \rho_{2} = \frac{{\rho_{b}^{2} }}{{\rho_{1} }} \hfill \\ c_{2} = c_{1} \hfill \\ \end{gathered} $$

$\rho_{1}$, $\rho_{2}$, $c_{1}$, and $c_{2}$ are the density and speed of sound of materials 1 and 2. $\rho_{b}$ and $c_{b}$, are the density and speed of sound of substrate. $R_{1}$ and $R_{2}$ are the inner and outer radius of the cloak and $r$ is the distance to the cylinder axis.

According to Eq. (1), it is impossible to obtain materials with this property in natural materials. Also, the material properties in the cloaking layers change with radial coordinates. However, this cloaking is composed of materials with anisotropic density and bulk scalar modulus. This research will examine the possibility of constructing acoustic cloaks using available natural or engineered materials. In the following, we will introduce the proposed cloaking and schematics of the structure. There two multilayer and composite lattice structures have been examined in detail along with the obtained results.

## Acoustic cloak based on multilayer cylindrical structure

This study introduces a multilayer cylindrical cloaking structure consisting of two alternating materials. This paper aims to obtain a suitable acoustic structure to design an applicable cloak for concealing various objects of any shape inside cloak area. Here, the first step is to choose the right material for the acoustic cloaking. According to the effective medium theory for the design of the cloaking, we must consider some points, firstly, the mass density of the substrate must be greater than the effective mass density of the acoustic cloaking. Second, the bulk modules of the substrate must be greater than the effective bulk modules of the cloaking. Considering these points, the proposed acoustic cloaking material has been selected. Also, theoretically, acoustic cloaks can be designed with acoustic metamaterials. However, the properties of acoustic cloaks are so severe in some areas that they cannot be made with natural materials. So, we have used two-dimensional inertial acoustic cloak design method based on coordinate transformation for mapping in radial directions. By dividing the cloak into small parts using Eq. (1) obtained from coordinate transformation, transverse anisotropy is removed and radial anisotropy remains. So as a result, we will have an anisotropic density shell. The proposed schematic is a shell consisting of an alternating multilayer structure. The multilayer cloak comprises water and the Methyl Nonafluorobutyl Ether (MNE). Methyl Nonafluorobutyl Ether (MNE) is a mild organic solvent solution widely used in cosmetic compositions. In addition, the low surface tension and high boiling point of MNE make it a cleaning solvent and lubricant. MNE offers popular properties such as non-flammable, colorless, non-toxic, relatively low vapor pressure, low odor, non-corrosive and transparent liquid that allows it to be used in various pharmaceutical and industrial applications^37^.

To achieve the phenomenon of acoustic cloaking using available materials, it is clear that we must start with a soft host matrix containing isotropic density. Therefore, matrix and scatterers should have high contrast in acoustic parameters. So, to satisfy these conditions, the combination of MNE and water forms a multi-layered concealment shell in the MNE substrate. The mass densities of MNE and water are $\rho_{MNE} = 1501$ kg/m^3^ and $\rho_{water} = 1482$ kg/m^3^, respectively, and their corresponding sound velocities are $c_{MNE} = 584$ m/s and $c_{water} = 998$ m/s. Due to the characteristics of the acoustic parameters of MNE and water that do not have radial dependence, the introduced cloak can be simpler to fabricate compared to other multi-layered cloaks.

**Figure 1:**
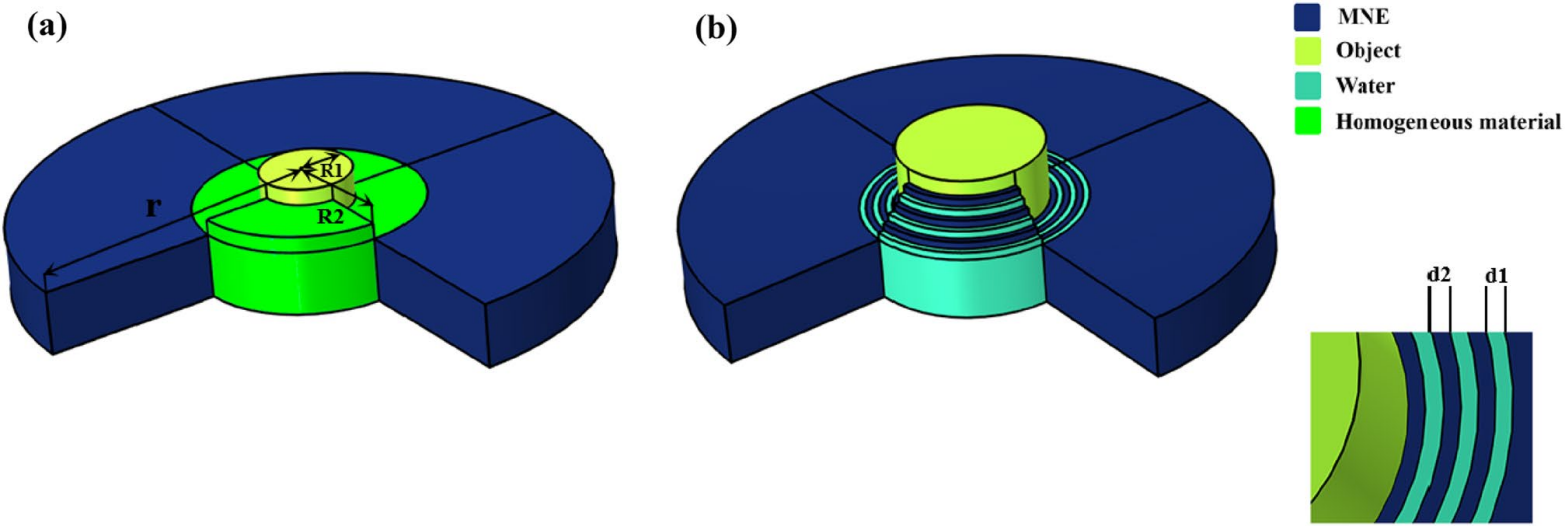
(**a**) Schematic of homogeneous cloak composed of anisotropic materials in MNE substrate and (**b**) schematic of a multilayer cloaking consisting of water and MNE in the MNE substrate.

The proposed cloaking structure is shown schematically in Fig. 1a, where the one-dimensional alternating structure is transformed into a circular shell to hide any rigid object with any shape inside it. According to Fig. 1 R1 and R2 are the inner and outer radii of the multilayer cloaking and r is the total radius of the structure. In order to better understand how the proposed cloaking works, a cylindrical single layer cloak structure of homogeneous materials with anisotropic properties is introduced in the MNE substrate. Anisotropic parameters of homogeneous materials include effective density and effective bulk modulus. By replacing $\rho_{b} = 1501$ kg/m^3^, R1 = 15 mm, R2 = 35 mm and r (related to cylindrical coordinates) in relation (1), the anisotropic variables of materials 1 and 2, which are dependent on x and y, are obtained. Next, by replacing the obtained variables (1) in (2)

2 $$ \begin{gathered} \rho_{Tangential} = \frac{{2\rho_{1} \rho_{2} }}{{\rho_{1} + \rho_{2} }} \hfill \\ \rho_{Normal} = \frac{{\rho_{1} + \rho_{2} }}{2} \hfill \\ \end{gathered} $$

the effective density is calculated. It should be noted that in relation (2), $\rho_{Tangential}$ shows the effective density along the layers and $\rho_{Normal}$ shows the effective density perpendicular to the layers. Also, by replacing the parameters in the following relation

3 $$ \begin{gathered} K_{1} = \rho_{1} c_{1}^{2} \hfill \\ K_{2} = \rho_{2} c_{2}^{2} \hfill \\ K = \frac{{2K_{1} K_{2} }}{{K_{1} + K_{2} }} \hfill \\ \end{gathered} $$

the effective bulk modulus K will be calculated. Here K_1_ is the bulk modulus of material 1 and K_2_ is the bulk modulus of material 2.

A schematic of this cloaking shell is shown in Fig. 1b. As shown in Fig. 1b R1 and R2are the inner and outer radii of the cylindrical single-layer cloaking, respectively, and r is the total radius of the structure. This paper focuses on the kHz range corresponding to the millimeter size of the structure parameters such as R1 = 15 mm, R2 = 35 mm and r = 80 mm, which is the area used in acoustic cloakings. It also prevents high GHz frequencies at which structural relaxation processes occur in the MNE polymer.

One of the methods to achieve the material properties for the acoustic cloaking mentioned in (1) is the use of layers consisting of two alternating fluids that change the effective density and modulus of the mass. According to^38^, the practical realization of Eq. (1) conditions is impossible due to the absence of matter with these properties. Also, the experimental realization of conditions (1) is impossible to construct according to the obtained dimensions. However, the multilayer structure, overcoming the mentioned problems, results in acoustic cloaking. This paper uses 15-layer and 30-layer models where d1 is the thickness of the water layer and d2 is the thickness of the MNE layer. In an alternating system, the bulk modulus does not depend on the lattice type. The effective bulk modulus can be determined from its reciprocal bulk average. Therefore, for a one-dimensional multilayer system consisting of materials 1 and 2, it is shown that^38^

4 $$ \frac{1}{{k^{*} }} = \frac{1}{{d_{1} + d_{2} }}\left\lceil {\frac{{d_{1} }}{{k_{1} }} + \frac{{d_{2} }}{{k_{2} }}} \right\rceil $$

Here, K1 and K2 are the Bulk module of materials 1 and 2, respectively, and d1 and d2 are the lengths of layers 1 and 2. Effective sound velocity can be obtained by extending the power of second-order trigonometric functions. According to Equations $k = \rho c^{2}$, $c_{ \bot }$ and $c_{\parallel }$ the diagonal components of the velocity tensor are propagated along with vertical and parallel directions^38^.

5 $$ c_{ \bot }^{2} = \frac{{k^{ * } }}{{\rho_{ \bot } }},c_{\parallel }^{2} = \frac{{k^{ * } }}{{\rho_{\parallel } }} $$

The following equation calculates $\rho_{ \bot }$ and $\rho_{\parallel }$,

6 $$ \rho_{ \bot } = \frac{1}{{d_{1} + d_{2} }}(d_{1} \rho_{1} + d_{2} \rho_{2} ),\begin{array}{*{20}c} {} & {} & {} & {} \\ \end{array} \frac{1}{{\rho_{\parallel } }} = \frac{1}{{d_{1} + d_{2} }}(d_{1} /\rho_{1} + d_{2} /\rho_{2} ) $$

which show the diagonal components of the effective dynamic mass density tensor. Next, we solve the Helm-Holtz equation for the total acoustic pressure at the operating frequency f = 29 kHz according to the following equation,

7 $$ \nabla .( - \rho^{ - 1} \nabla p_{t} ) - \frac{{\omega^{2} p_{t} }}{k} = 0 $$

Here, $\rho^{ - 1}$ is a tensor for the anisotropic material, the Bulk modulus $k = \rho c^{2}$ for the isotropic material, and $P_{t}$ is the total acoustic pressure. To describe an incident plane wave traveling in the x-direction, a background field $P_{b}$ is defined $e^{{ - k_{b} x}}$ as $k_{b} = 2\pi f/c_{b}$ the propagation constant in the background medium. The equation is solved for the scattered field $p_{s}$, using the definition

8 $$ p_{t} \equiv p_{b} + p_{s} $$

## Results and discussions of the multilayer cloak

**Figure 2:**
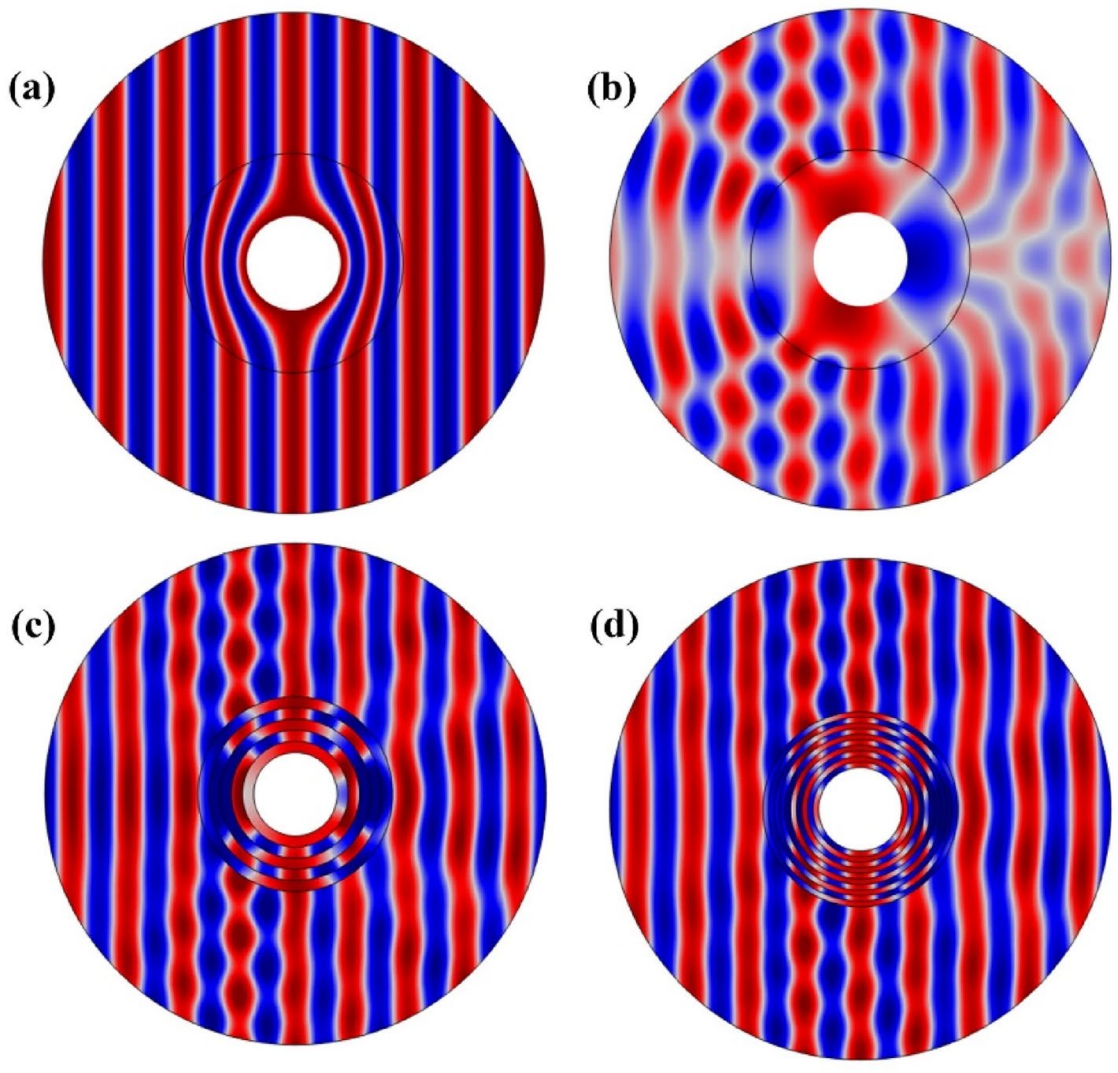
The total acoustic pressure field calculated by the FEM method at f = 29 kHz for (**a**) homogeneous cloak with anisotropic materials, (**b**) structure without cloak in MNE substrate, (**c**) multi-layer cloaking with 5 layers and (**d**) multi-layer cloaking with 10 layers.

To investigate the performance of the multilayer cloaking, we performed multiple scattering simulations using the COMSOL Multiphysics software. The total acoustic pressure for the four different models can be seen in Fig. 2. Figure 2b (top right) shows the pressure field without the cloak when the cylinder is surrounded only by the MNE. The incident pressure wave is scattered in all directions and significantly affects the cylinder. In Fig. 2a (top left), a homogeneous cloak is used. Like the incident wave, the output wave is unchanged to the cloak, and it is impossible to detect the object. Figure 2c,d show multi-layer cloaking structure with 5-layer and 10-layer cloaks, respectively. The thickness of the layers of the 5-layer structure is d1 = d2 = 2 mm and the 10-layer is d1 = d2 = 1 mm. The cloaking effect is evident in Fig. 2c and (d), but a structure 10-layers can be considered almost near homogeneous cloak. Nevertheless, we can analyze the cloaking effect as a function of the number of layers used to make the acoustic cloaking.

According to Fig. 2c,d, compared to Fig. 2b, which has no cloaking shell, the acoustic waves are reconstructed after passing through the object. From the pressure distributions Fig. 2c,d, we can conclude that the level of cloaking depends on the type of layer design and the properties of the material can have a greater impact on the acoustic cloak than the geometric parameters in this optimized model.

**Figure 3:**
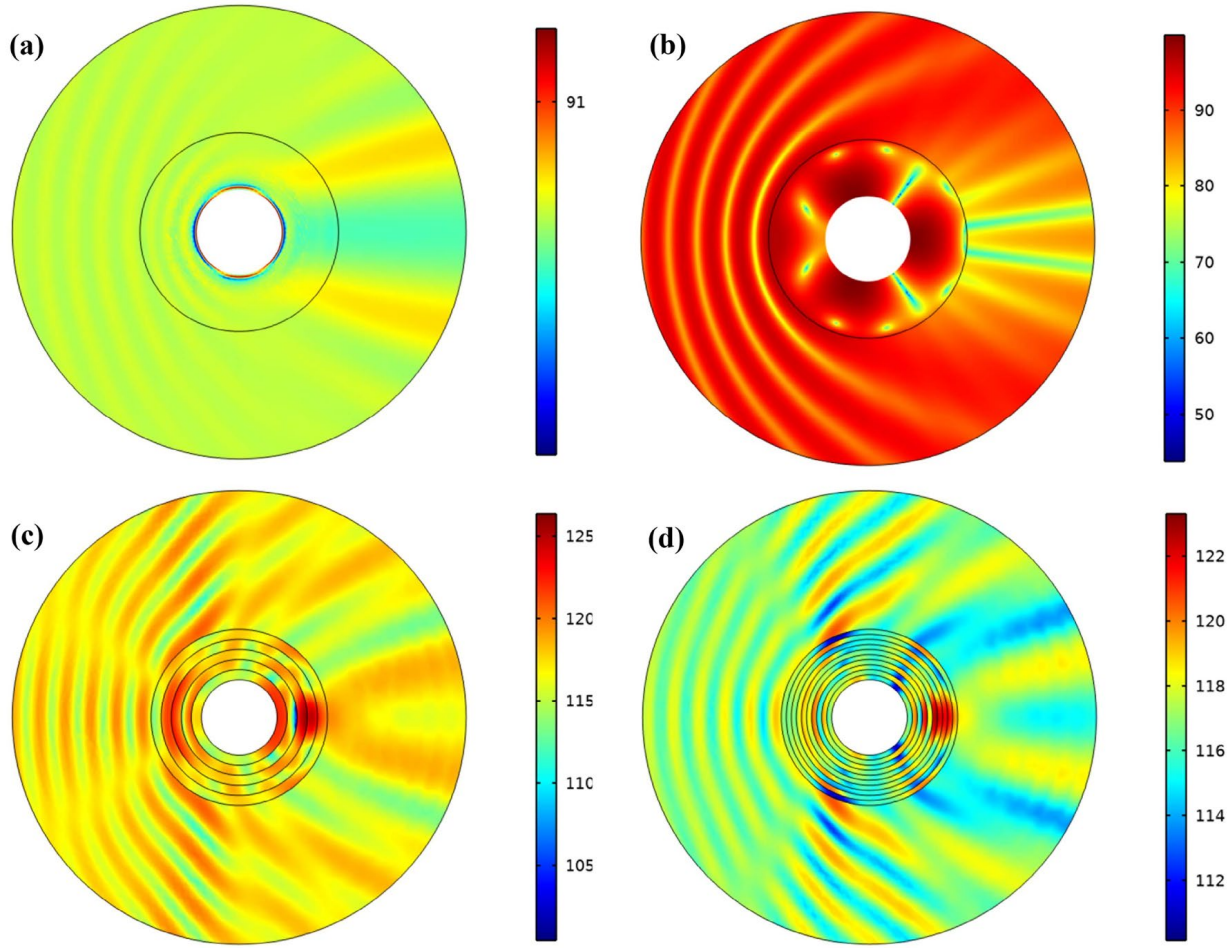
The total sound pressure level for (**a**) homogeneous cloak structure with anisotropic materials, (**b**) no cloak structure and multi-layers cloaking structure with (**c**) 5 layer and (**d**) 10 layers.

Figure 3 shows the total sound pressure level for similar cases. When the cloaking is not used according to Fig. 3b, the shadow area behind the cylinder is easily visible and the pressure peaks on the side where the wave sinks. In the homogeneous cloak (Fig. 3a), pressure changes are not visible. It can be easily seen from Fig. 3 that the structure introduced in this section is somewhat close to cloak the homogeneous structure. Given the multilayer structure introduced, it can be concluded that to have an acoustic cloaking, ultra-high-density materials are not required along with very low-density materials and as a result, cloaking can be achieved with available natural materials.

**Figure 4:**
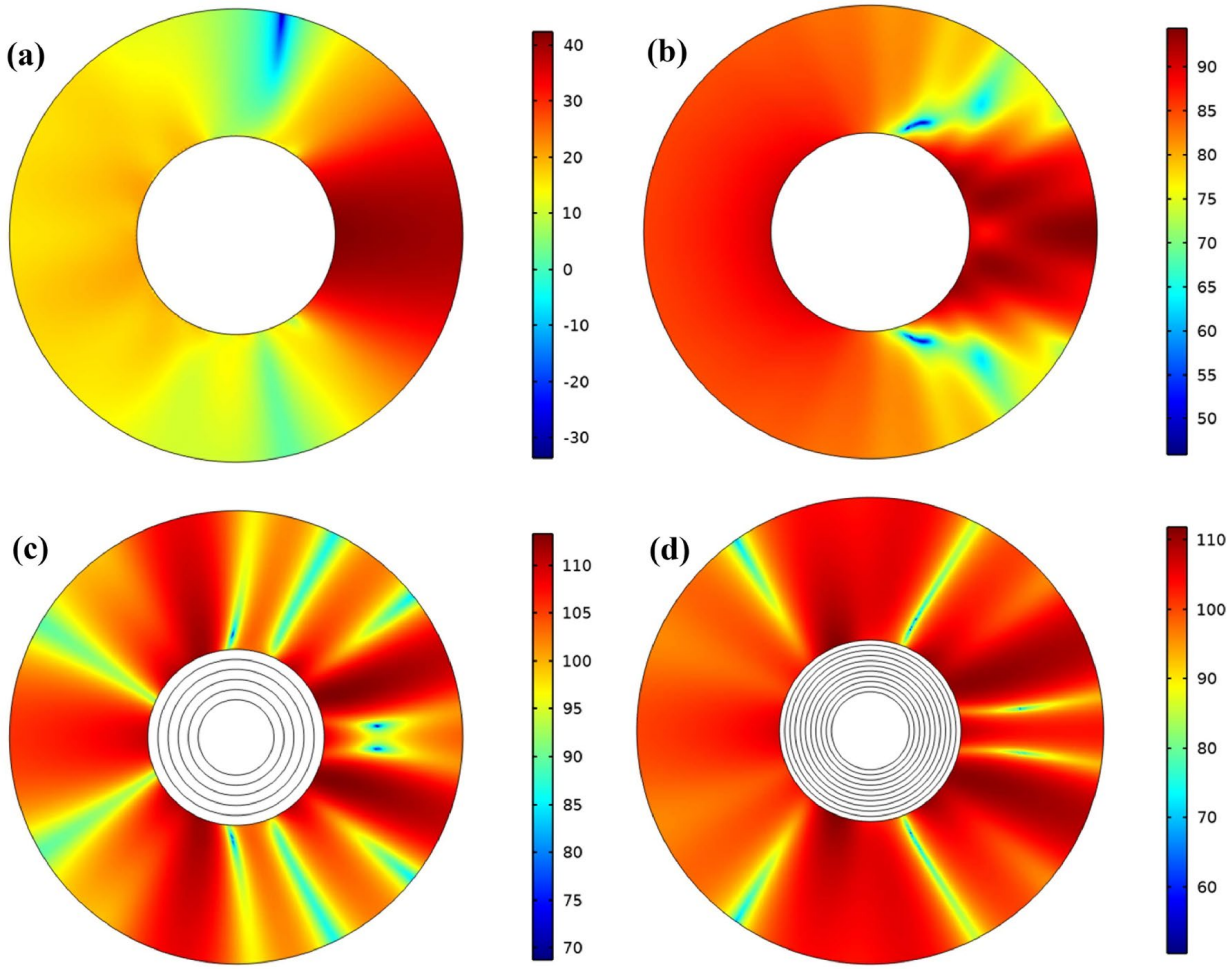
The scattering pressure level for (**a**) homogeneous cloak, (**b**) no cloak structure and multi-layers cloaking with (**c**) 5 layer and (**d**) 10 layers.

Figure 4 shows the scattering pressure level for all four models to better understand the designed cloak. A significant difference in the scattered field can be observed for the four cases with just a simple look at the scattered sound pressure level. Therefore, the reduction of the scattered sound pressure level indicates the tendency of the cloaking to hide and approach the homogeneous model. Here the scattered sound pressure field shows clearer evidence that the multilayer model dramatically reduces the scattered pressures in the direction of the plane wave input.

We are looking for scatter on the source side and behind the object. In a 10-layer structure, most of the acoustic waves that reach the object are propagated forward through the lattice structure, minimizing dorsal scattering, which is different from the no cloak structure in which the waves propagate in all directions.

## Acoustic cloak based on composite lattice structure

**Figure 5:**
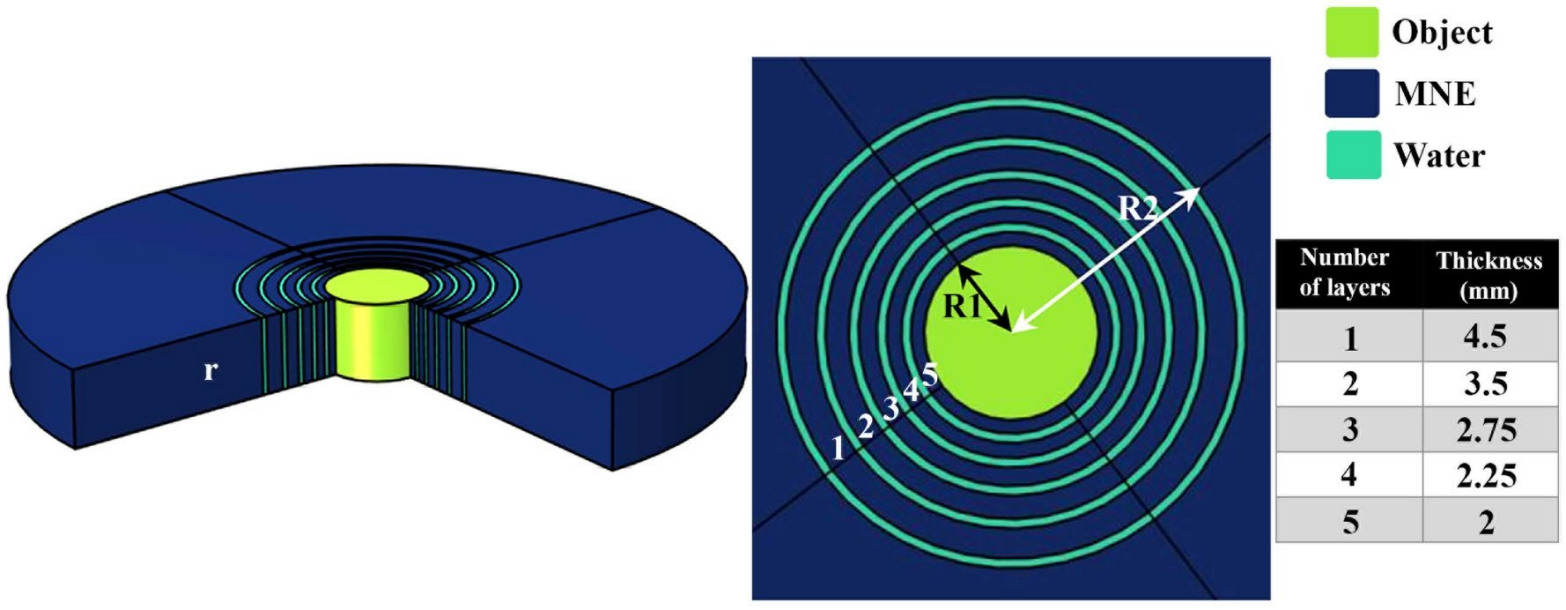
Schematic of composite lattice cloaking composed of natural materials in MNE substrate. The introduced cloaking structure consists of water and MNE layers. The water layers have a constant thickness of 1.4 mm and the thickness of the numbered MNE layers is shown in the figure.

Here, a multilayer cylindrical structure consisting of two materials is introduced as a composite grid. The purpose of this section is to improve the performance of the acoustic cloaking phenomenon and to design an easy-to-apply shell for cloaking. The proposed schematic of a multilayer shell is different from the structure described in the previous section. The structure is composed of layers of water and MNE, with the difference that the thickness of the layers of water is constant throughout the structure, but the thickness of the layers of MNE gradually decreases with a certain rhythm. The proposed cloaking is shown schematically in Fig. 2a, where the one-dimensional composite structure is transformed into a circular shell to hide any hard object with any shape inside it. According to Fig. 5, R1 and R2 are the internal and external radii of the composite lattice cloak and also r is the overall radius of the structure. Also, according to Fig. 5, the thickness of the layers of the proposed cloaking is shown.

In the following, we will review the results of the proposed coverage according to the steps taken for the previous structure. It should be noted that to better understand how the proposed cloaking structures work and the effect of increasing the layers on the performance of the proposed cloaking introduced in the previous section, as well as the comparability of all three homogeneous structures with anisotropic properties, multilayer structure and composite structure introduced in this section, The simulations performed for all three items are repeated as in the previous section.

## Results and discussions of the composite lattice structure

**Figure 6:**
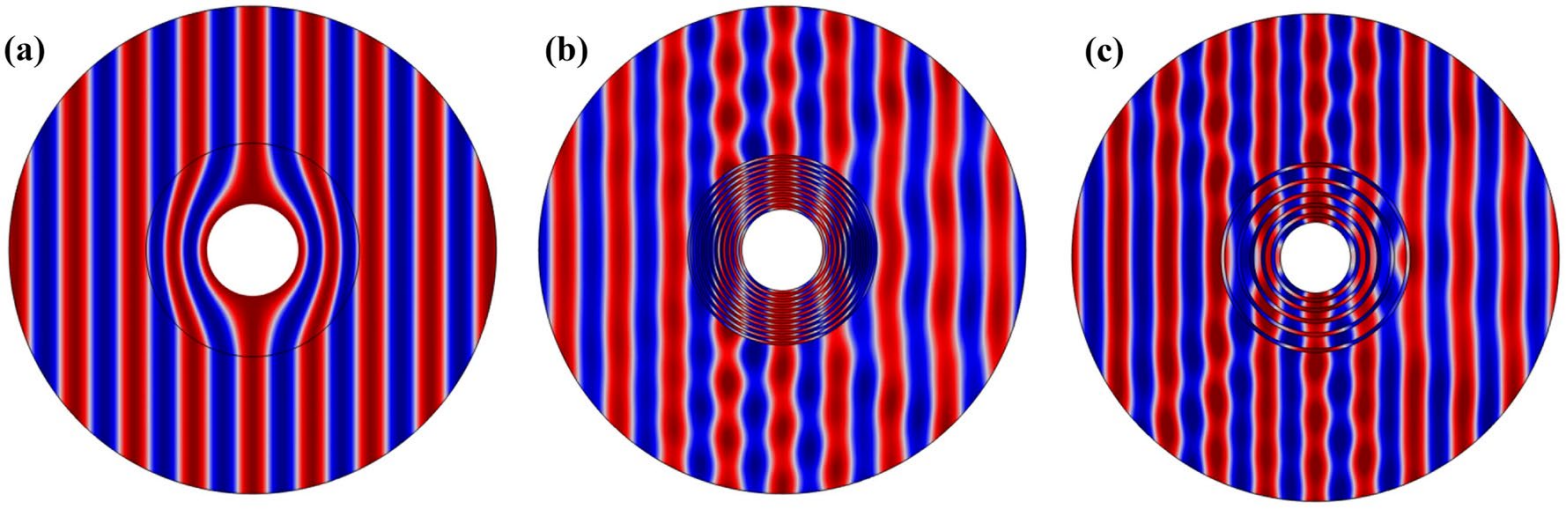
The total acoustic pressure field at f = 29 kHz for (**a**) homogeneous cloak with anisotropic materials, (**b**) multi-layer cloaking with 20 layers and (**c**) composite lattice cloaking with 10 layers of variable thickness.

To evaluate the performance of the composite cloaking, we have performed multiple scattering simulations for the composite structure, the multilayer structure with 20 layers and the homogeneous structure with anisotropic properties. The total acoustic pressure for all three modes is shown in Fig. 3. Figure 6a shows the acoustic pressure field of a homogeneous structure, when the cylinder is surrounded only by a material with anisotropic properties. The incident pressure wave is non-scattered in all directions and is significantly affected by the anisotropic cover and no reflections or shadows are created. It is difficult to detect an object in this type of cloaking, but it is impossible to find materials that have such properties in materials found in nature. Figure b shows the acoustic pressure field for a multilayer structure (20 layers consisting of water and MNE with the same thickness equal d1 = d2 = 0.5 mm). Considering the total acoustic pressure curve of Fig. 6 compared to Fig. 2c,d, we conclude that by increasing the number of layers from a certain limit, the total acoustic pressure field will remain unchanged and the cloakings will have a similar performance. Figure 6c show the acoustic pressure field of the composite structure. Due to the acoustic pressure field output, we see an improvement in the performance of the composite cloaking compared to the multilayer cloaking.

In this cloaking, by gradually increasing the MNE layers, we have increased the effect of the substrate on the acoustic cloaking phenomenon. According to the pressure distribution of Fig. 6b,c, we can conclude that the level of cloaking depends on the type of layer design and the properties of the material can have a greater impact on the acoustic cloaking than the geometric parameters in this optimized model.

**Figure 7:**
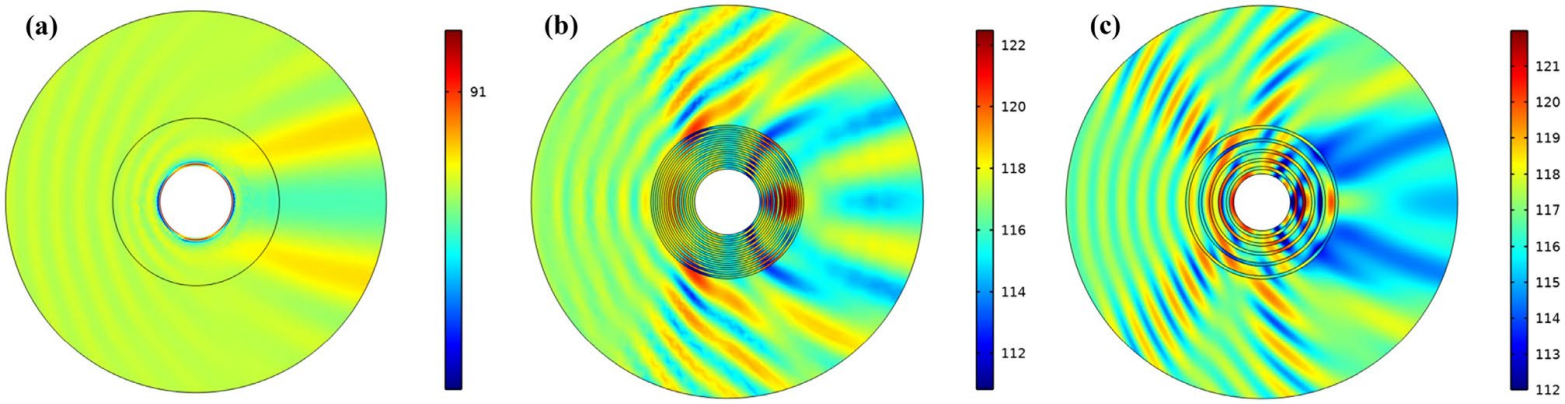
The total sound pressure level for (**a**) homogeneous cloaking, (**b**) multi-layers cloaking with 20 layer and (**c**) composite lattice cloaking.

The total sound pressure level is calculated similarly for all three items and is shown in the Fig. 7. According to Fig. 7a, which is related to the homogeneous cloak, it can be seen that the pressure changes at the inlet side are very small and we have the highest pressure behind the cylinder, which is a small amount compared to the proposed structures. When multi-layer cloaking (20 layers) is used (Fig. 7b), we see that increasing the number of layers compared to Fig. 3c, reduces the total pressure level and the shadow area behind the cylinder has less pressure. Figure 7c shows the total acoustic pressure level of the composite structure, which has decreased compared to the multilayer structure. According to Fig. 7c, changing the composition of the layers next to each other relative to the multilayer state causes the structure to be closer to a homogeneous cloaking.

**Figure 8:**
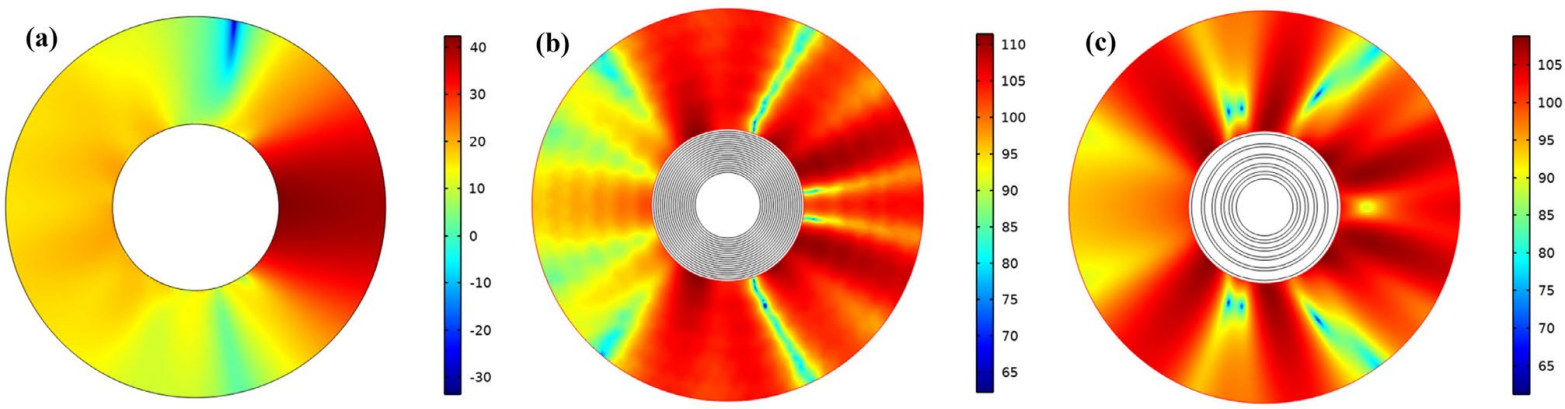
The scattering pressure level for (**a**) homogeneous cloak, (**b**) multi-layers cloaking with 20 layer and (**c**) composite lattice cloaking.

Similarly, multilayer cylindrical structure, Fig. 8 shows the scatter pressure level for all three states, which according to the scattered sound pressure level, the difference in the scattered field can be observed for all three states. The decrease in scattered sound pressure level indicates the tendency of the shell to cloaking and approach a homogeneous model. According to Fig. 8a, the scattered sound pressure field reduces the scattered pressures towards the input of the plane waves. Comparing Fig. 8b,c and considering that the dispersions are in the direction of the source and the back of the object, we find that the pressure level in the introduced composite structure is lower than the multilayer structure and the dispersions in the inlet side are more uniform than the structure multi-layered. In both the multilayer and composite structures, compared to the no-cloak structure, when the acoustic waves reach the object, they propagate forward through the acoustic cloak and the back scattering is minimized, while in the no-cloak structure, the waves propagate in all directions.

**Figure 9:**
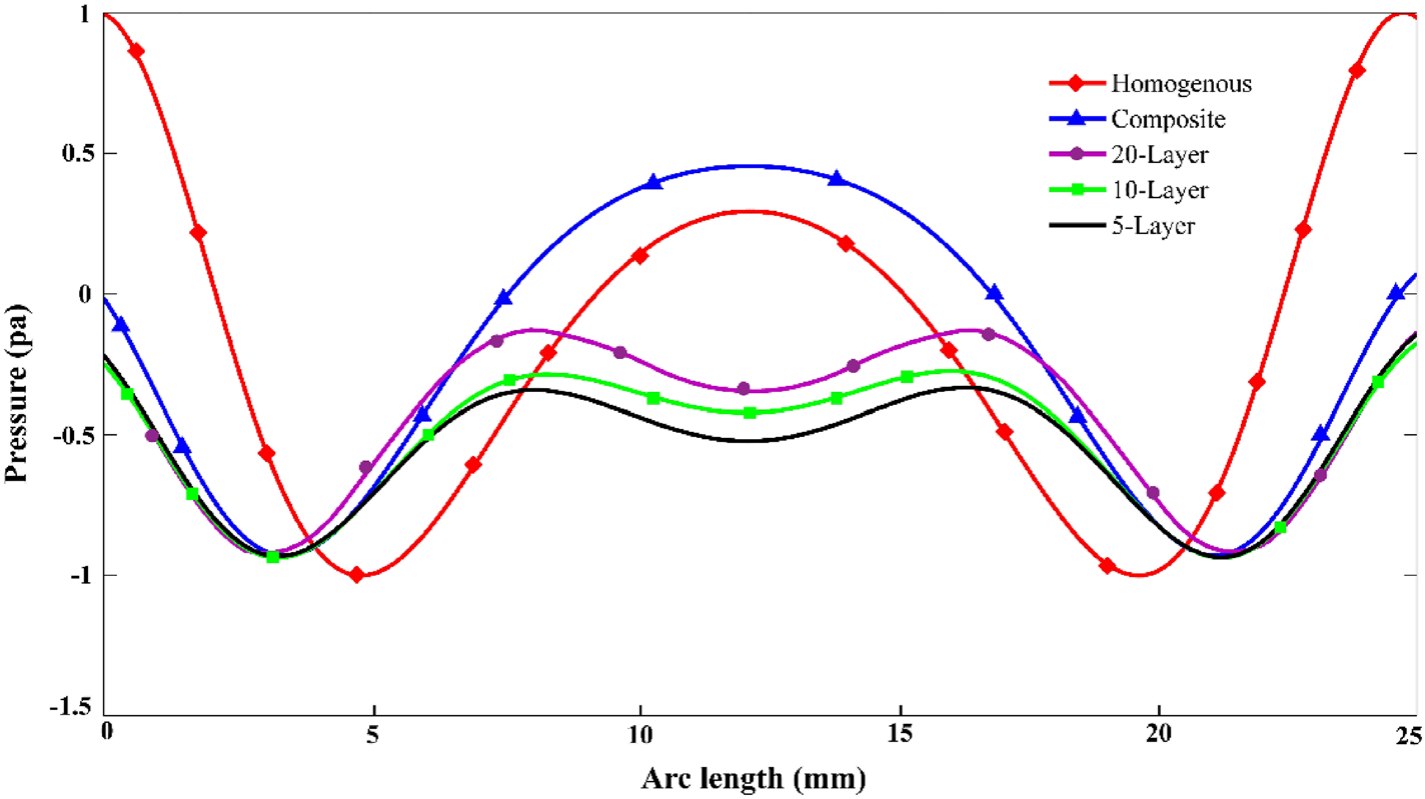
The background pressure field curve calculated along the boundary of cloaking structure for homogenous, composite, multi layer’s structure.

To determine the quality of the cloak designed by the multilayer and composite model compared to the homogeneous model, the performance of the cloakings by calculating the total acoustic pressure along the cloaking structure boundary is shown in Fig. 9. According to the Fig. 9, we see that the background pressure field curve for a 20-layer and composite cloaking to be approximately close to the homogeneous coverage curve. The main difference between the introduced structures and the homogeneous structure is that the homogeneous structure introduced in both X‌ and Y directions has anisotropy, However, the introduced structures, due to the fact that they are made of natural materials, fulfill the cloaking property only in the direction of the input wave. Introduced structures have been optimized at 29 kHz where the acoustic cloaking can be seen in frequency below 29 kHz.

The theory used in this research is effective medium theory. In fact, when each layer has the right density and volume modulus, the structure can act as an effective medium. Full-wave simulations with finite element method have been performed to show the reflectance and flexural properties of the impact wave front to the structure. The cloaked object may not be compatible with the external medium or may be completely compatible, however it can cloak a wide range of materials. In this design, we tried to design a shell that the hidden object has no dependence on the type of material, that is, any object with any material can be hidden. Note also that the shell is designed to work in a wide frequency range, at a high level determined by the thickness of the layer. Here we show the possibility of achieving an acoustic cloaking with ordinary isotropic materials instead of acoustic metamaterials with complex structural components. When the thickness of each layer is much smaller than the impact wavelength, the microstructure system can be considered as a single anisotropic material based on the effective medium approximation.

## Conclusions

This paper shows that acoustic cloaking shells are possible using multilayer and composite structures with two isotropic acoustic materials. Therefore, we do not need metamaterials to design these types of shells. It has also been shown that acoustic parameters have no radial dependence, so they are easier to fabricate than other multilayer cloakings. The proposed cloaking has no incident angle limitations and, as another significant advantage compared to electromagnetic cloakings, has acceptable performance in a wide range of wavelengths. In addition, the materials used in this cloaking are natural and available. According to the introduced structures, it was concluded that to have an acoustic cloaking, high-density materials are not required along with ultra-low-density layers, and acoustic cloaking can also be achieved with available natural materials.

## Data Availability

The data that support the findings of this study are available from the corresponding author upon reasonable request.
